# All‐cause versus cause‐specific excess deaths for estimating influenza‐associated mortality in Denmark, Spain, and the United States

**DOI:** 10.1111/irv.12966

**Published:** 2022-02-23

**Authors:** Sebastian S. S. Schmidt, Angela Danielle Iuliano, Lasse S. Vestergaard, Clara Mazagatos‐Ateca, Amparo Larrauri, Jan M. Brauner, Sonja J. Olsen, Jens Nielsen, Joshua A. Salomon, Tyra G. Krause

**Affiliations:** ^1^ Department of Infectious Disease Epidemiology Statens Serum Institut Copenhagen Denmark; ^2^ Influenza Division Centers for Disease Control and Prevention Atlanta GA USA; ^3^ National Centre of Epidemiology, CIBER Epidemiología y Salud Pública (CIBERESP) Carlos III Health Institute Madrid Spain; ^4^ Department of Computer Science University of Oxford Oxford UK; ^5^ Health Emergencies Program World Health Organization Regional Office for Europe Copenhagen Denmark; ^6^ Department of Medicine Stanford University Palo Alto CA USA

**Keywords:** all‐cause deaths, cause of death, EuroMOMO, excess mortality, influenza‐associated mortality

## Abstract

**Background:**

Seasonal influenza‐associated excess mortality estimates can be timely and provide useful information on the severity of an epidemic. This methodology can be leveraged during an emergency response or pandemic.

**Method:**

For Denmark, Spain, and the United States, we estimated age‐stratified excess mortality for (i) all‐cause, (ii) respiratory and circulatory, (iii) circulatory, (iv) respiratory, and (v) pneumonia, and influenza causes of death for the 2015/2016 and 2016/2017 influenza seasons. We quantified differences between the countries and seasonal excess mortality estimates and the death categories. We used a time‐series linear regression model accounting for time and seasonal trends using mortality data from 2010 through 2017.

**Results:**

The respective periods of weekly excess mortality for all‐cause and cause‐specific deaths were similar in their chronological patterns. Seasonal all‐cause excess mortality rates for the 2015/2016 and 2016/2017 influenza seasons were 4.7 (3.3–6.1) and 14.3 (13.0–15.6) per 100,000 population, for the United States; 20.3 (15.8–25.0) and 24.0 (19.3–28.7) per 100,000 population for Denmark; and 22.9 (18.9–26.9) and 52.9 (49.1–56.8) per 100,000 population for Spain. Seasonal respiratory and circulatory excess mortality estimates were two to three times lower than the all‐cause estimates.

**Discussion:**

We observed fewer influenza‐associated deaths when we examined cause‐specific death categories compared with all‐cause deaths and observed the same trends in peaks in deaths with all death causes. Because all‐cause deaths are more available, these models can be used to monitor virus activity in near real time. This approach may contribute to the development of timely mortality monitoring systems during public health emergencies.

## 1 INTRODUCTION

Globally, influenza has been estimated to be associated with up to 646,000 respiratory deaths annually with significant seasonal variation.^1^ Furthermore, influenza pandemics can result in much greater mortality than observed. The 1918 influenza pandemic resulted in approximately 50 million deaths and reduced the life‐expectancy in the United States by 12 years.^2^ However, the risks posed by new strains of respiratory viruses have never been more salient.^3^ The ongoing COVID‐19 pandemic highlights a need for robust and timely mortality estimates for assessing risks and prioritizing public health interventions during seasonal influenza epidemics and during novel virus pandemics.

There are many different methods used for estimating the influenza‐associated mortality.^1^, ^4^, ^5^, ^6^, ^7^ One important distinguishing factor is the use all‐cause mortality compared with selected cause‐specific mortality data. All‐cause mortality data are, by definition, multicausal, and it is only possible to associate excess mortality to a specific underlying cause (e.g., influenza) by assessing the correlation over time with specific indicators of that specific cause. Because of this multicausality, all‐cause excess mortality may overestimate the true number of deaths associated with one cause.^8^ However, during winter seasons in temperate zones, increases in all‐cause mortality are often observed, and the circulation of influenza has been historically shown to be the main seasonal driver of this excess mortality, although other factors (such as extreme temperatures and other respiratory viruses) may contribute as well.^8^, ^9^, ^10^ Furthermore, all‐cause mortality data are readily available in many countries with a short delay and can be used for weekly monitoring to inform timely risk assessments of a wide range of threats.^11^

Cause‐specific mortality data, on the other hand, can be more disease‐specific and may provide estimates that are more accurate. However, the categories commonly used for estimating the influenza‐associated mortality (such as respiratory diseases) are also multicausal and contain many causes unrelated to events of interest for monitoring of influenza‐associated mortality.^1^ Due to the higher specificity, models based on cause‐specific data (e.g., respiratory diseases) tend to lose sensitivity and may underestimate the total number of deaths associated with influenza.^1^, ^8^, ^12^, ^13^, ^14^ Furthermore, processes to clean, code underlying and contributing death causes, and to validate the data are currently causing up to 2 years of delays in most countries and potentially even longer delays during pandemics. For these reasons, cause‐specific mortality data may be less suitable and less timely for real‐time monitoring purposes.

The true influenza mortality burden is likely to be between these two outcome data sources used in estimation. Therefore, cause‐specific mortality data are valuable for validating excess mortality estimates derived from all‐cause mortality data. However, few direct comparisons between the estimates obtained using these two data sources have been made.

Emerging threats like the COVID‐19 pandemic highlight the utility of monitoring all‐cause mortality in real time. By the time global guidance for coding deaths due to COVID‐19 was released on April 16, 2020, 131,034 global deaths were already reported.^3^, ^15^ However, it has become apparent through estimates of all‐cause excess mortality that the reporting is likely an underestimate.^11^, ^16^

Since 2009, the European network for monitoring of excess mortality for public health action, EuroMOMO, has monitored weekly all‐cause mortality in up to 24 participating European countries and provided pooled estimates of excess mortality (observed deaths minus baseline deaths), using the EuroMOMO model.^11^, ^17^, ^18^, ^19^ We examined the differences between using all‐cause and select cause‐specific mortality data for estimating mortality related to influenza. We did this by applying the EuroMOMO model to (i) all‐cause, (ii) pneumonia and influenza (P&I), (iii) respiratory and circulatory, (iv) respiratory, and (v) circulatory mortality data. We compared the weekly and seasonal cumulative excess mortality estimates using data from Denmark, Spain, and the United States for the 2015/2016 and 2016/2017 seasons and compared these estimates with official estimates from the national public health authorities in these countries.

## 2 MATERIAL AND METHODS

### 2.1 Data sources

#### 2.1.1 Mortality data

To estimate the weekly mortality attributable to influenza, we used mortality data by age group (0–64, 65–74, and ≥75 years) from the 2010/2011 through the 2016/2017 season. Deaths were categorized using the International Classification of Diseases, 10th Revision (ICD‐10) codes. We focused on underlying causes of death categorized as all‐cause (A00‐Y99), diseases of the circulatory system (I00‐I99), diseases of the respiratory system (J00‐J99), influenza (J9‐J11), and pneumonia (J12‐J18). For each mortality record, the primary underlying cause was listed, defined as “the disease or injury which initiated the train of morbid events leading directly to death, or the circumstances of the accident or violence which produced the fatal injury” as specified by ICD‐10.^20^

For Denmark, the mortality data were obtained as individual notifications with the underlying cause of death, dates of deaths, and dates of birth from the Danish Civil Registry. For the United States, the mortality data were obtained as weekly aggregated age group‐specific data from the National Center for Health Statistics (NCHS). For Spain, the mortality data were obtained as weekly aggregated age group‐specific data from computerized civil registers covering 92% of the total Spanish population through the Centro Nacional de Epidemiología, Instituto de Salud Carlos III (CNE‐ISCIII).

#### 2.1.2 Mortality rates—Population data

For Denmark and Spain, the population data were downloaded from Eurostat in Week 5/2020.^21^ For the United States, the population data were obtained from the United States Census Bureau.^22^ Based on the estimated number of deaths, mortality rates were calculated using national population data as of January 1 every year and linearly interpolated through the year. We interpolated population data provided by each country on December 31 of each study year to obtain weekly population counts using the following formula:

$$y=y_{1}+\left(x-x_{1}\right)\frac{y_{2}-y_{1}}{x_{2}-x_{1}},$$

where *y* = population at a given week (e.g., Week 422,014), *X* = given time (e.g., Wednesday, Week 422,014), *y*_2_ = known population at time after *x* (e.g., Week 532,017), *y*_1_ = known population at time before time *x* (e.g., Week 12,013).

### 2.2 The EuroMOMO model

The model is a time‐series regression model using a Poisson distribution and corrected for overdispersion and ISO‐week* as the time unit and the number of weekly deaths as the dependent variable adjusting for time trends and seasonal variation typically used in real time.^17^ Some of the main outputs are total weekly number of deaths corrected for delay in registration when the model is used in real time, expected weekly number of deaths (baseline), weekly number of excess deaths (defined as observed number minus the expected number of deaths), and standard deviation around the baseline (*z* score) by all ages and stratified into age groups. For this project, we used this model without the adjustment for delay in death registration because the death data used were retrospective and were completely verified, cleaned, and processed. Correction for completeness of the data is typically completed when the data are being used in real time. The baseline was estimated based on the five preceding seasons (2010/2011 through 2014/2015) during the period of the year when it was assumed that additional factors that can lead to excess deaths are not likely to happen (primarily influenza and heat waves). These periods are relatively short compared with the whole of the series and are defined as “Spring” from Week 15 to Week 26 and “Autumn” from Week 36 to Week 45.

### 2.3 Analysis

#### 2.3.1 Arriving at the excess mortality estimates

The baseline for weekly mortality for season 2015/2016 and 2016/2017 was estimated by fitting the EuroMOMO model to the mortality data for the five preceding seasons. The weekly excess mortality estimates were calculated by subtracting the baseline mortality from the observed mortality. Stata 14 was used for statistical analyses.

#### 2.3.2 Weekly all‐cause excess mortality compared with excess cause‐specific

To evaluate differences in the weekly excess mortality estimates for all ages, we plotted the observed mortality rates and the baseline mortality rates for each cause of death and compared the timing of peak excess mortality periods for each country and the different disease causes. All estimates were converted to mortality rates per 100,000 population and adjusted for age according to the WHO World Standard Population.^23^

#### 2.3.3 Seasonal all‐cause excess mortality compared to excess cause‐specific

To evaluate differences by season, we summed the weekly excess mortality estimates—without truncating negative excess estimates to zero—within the entire period where the influenza season can possibly occur (Week 40 of 1 year through Week 20 of the following year) for season 2015/2016 and 2016/2017. We compared these estimates with the influenza‐associated mortality estimates published by the national public health authorities in Denmark, Spain, and the United States. To assess the relationship between the excess death estimates from different cause of death outcomes, we present the ratio of P&I excess deaths (referent) compared with the other causes of death that are commonly used.

## 3 RESULTS

### 3.1 Weekly all‐cause and cause‐specific excess mortality—The United States

**FIGURE 1:**
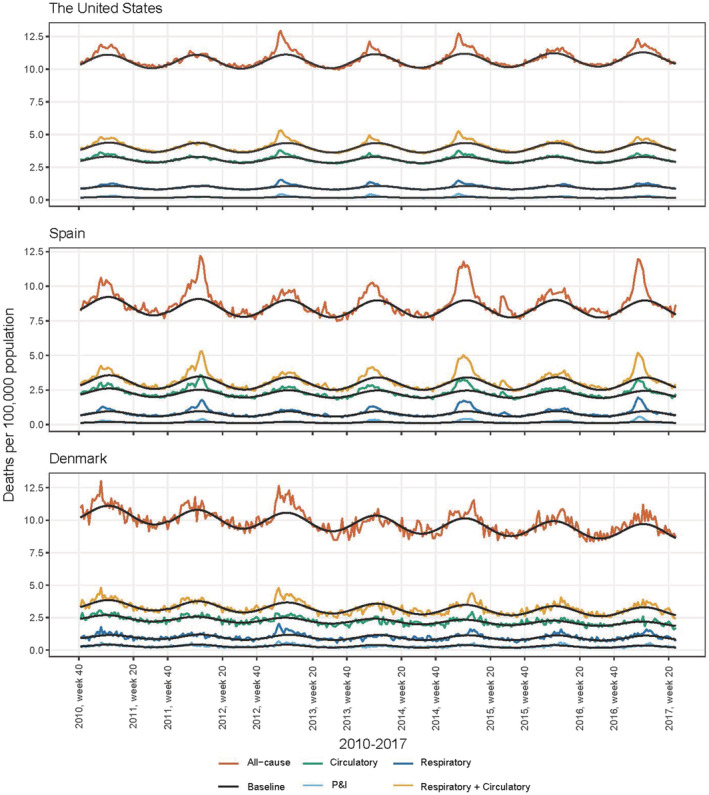
Weekly age‐adjusted mortality per 100,000 population for all‐cause, respiratory and circulatory, circulatory, respiratory, and pneumonia and influenza deaths, Week 40/2010 to Week 20/2017, in the United States, Spain, and Denmark. The modeled baseline is based on the EuroMOMO model. Excess mortality is defined as the observed mortality minus the baseline mortality. Note that anything before Week 40 of 2015 has been used to estimate the baseline, and therefore, the excess mortality will not be examined for that period

Within the possible influenza season (Week 40 to Week 20), there were distinct periods with excess mortality for all‐cause and cause‐specific mortality (Figure 1). For each year, the periods of excess mortality for all‐cause and the cause‐specific mortality were similar to each other in terms of when the excess mortality started, the timing of peaks, and the length of the period. The 2015/2016 season had little excess mortality across all‐cause and cause‐specific mortality, and the period with excess mortality started later compared with the 2016/2017 season.

### 3.2 Weekly all‐cause and cause‐specific excess mortality—Spain

Within the possible influenza season, there were distinct periods with excess mortality for all‐cause and cause‐specific mortality (Figure 1). The periods with excess mortality for circulatory mortality were similar to all‐cause mortality with respect to when the excess mortality started, the timing of peaks, and the length of the period. The periods with excess mortality for P&I and respiratory mortality tended to start later than all‐cause and circulatory mortality, but the peaks were synchronous. There was significantly more excess mortality in the 2016/2017 season than in the 2015/2016 season.

### 3.3 Weekly all‐cause and cause‐specific excess mortality—Denmark

Within the possible influenza season, we observed periods with excess mortality across all‐cause and cause‐specific mortality (Figure 1). However, it was more difficult to determine the periods of excess mortality due to the smaller population size. In particular, we observed distinct periods of excess mortality in respiratory and all‐cause mortality. Compared with all‐cause excess mortality, the respiratory and P&I excess mortality started later and were shorter in length but similar in the timing of peaks.

### 3.4 Seasonal all‐cause and cause‐specific excess mortality—The United States

For all ages, the mean excess mortality for the two seasons based on all‐cause, respiratory and circulatory, circulatory, respiratory, and P&I was 9.50, 4.30, 2.20, 2.09, and 1.01 per 100,000 population, respectively (Table 1). These estimates corresponded to 30,680, 12,429, 7094, 8182, and 3253 deaths, respectively. The excess mortality estimates for the 2016/2017 season were around three times as high as those for the 2015/2016 season. Influenza A (H1N1)pdm09 viruses predominated during the 2015/2016 season, whereas influenza A(H3N2) predominated during the 2016/2017 season (Table 1). The ratios between P&I mortality and the excess mortality from the other causes of death were all positive, and although they all increased slightly from the 2015/2016 to the 2016/2017 season, they were fairly uniform (Table 2). For example, all‐cause excess deaths were 9–10 time more each year than P&I excess deaths. When stratified by age groups, in the 2015/2016 season, the ratios were positive and increased for each cause of death category for the <65‐year and the 65‐ to 74‐year age groups. In the 2016/2017 season, they increased for the 65–74 and ≥75‐year age group.

**TABLE 1:** Cumulated all‐cause, respiratory and circulatory, circulatory, respiratory, and pneumonia and influenza excess mortality during the winter season (Week 40 to Week 20) and percent of influenza circulating subtypes by season 2015/2016 and 2016/2017, in the United States, Spain, and Denmark

The United States							
Season	Cause of death	All‐cause	Respiratory + circulatory	Circulatory	Respiratory	Pneumonia and influenza	Circulating types of influenza^a^
2015/2016	Age groups Excess mortality per 100,000 population (95% CI)						A(H1N1)pdm09 (57%) A(H3N2) (14%) B/Victoria (29%) ___
	0–64	5.2 (4.7; 5.8)	1.9 (1.7; 2.1)	1.4 (1.2; 1.6)	0.4 (0.3; 0.5)	0.2 (0.2; 0.3)	
	65–74	5.5 (2.5; 8.5)	5.4 (3.6; 7.2)	4.0 (2.6; 5.4)	1.5 (0.5; 2.5)	1.1 (0.7; 1.5)	
	≥75	−7.6 (−22.2; 7.1)	−6.9 (−15.9; 2.2)	−11.1 (−17.6; −4.5)	4.5 (1.0; 8.1)	2.4 (1.2; 3.7)	
	All ages	4.7 (3.3; 6.1)	1.7 (0.9; 2.4)	0.9 (0.3; 1.4)	0.8 (0.5; 1.1)	0.5 (0.4; 0.6)	
2016/2017	Age groups Excess mortality per 100,000 population (95% CI)						A(H3N2) (76%) A(H1N1)pdm09 (2%) B/Yamagata (22%)
	0–64	4.9 (4.3; 5.4)	1.6 (1.4; 1.8)	1.3 (1.1; 1.4)	0.3 (0.2; 0.4)	−0.0 (−0.1; 0.0)	
	65–74	19.9 (16.9; 22.9)	11.0 (9.4; 12.6)	6.1 (4.7; 7,5)	4.9 (4.0; 5.9)	1.8 (1.5; 2.2)	
	≥75	128.8 (114.8; 143.0)	70.7 (62.6; 78.8)	28.6 (22.5; 34.6)	42.2 (39.1; 45.4)	21.5 (20.2;22.9)	
	All ages	14.3 (13.0; 15.6)	6.9 (6.2; 7.6)	3.5 (3.0; 4.0)	3.4 (3.1; 3.6)	1.6 (1.4; 1.7)	

SPAIN							
Season	Cause of death	All‐cause	Respiratory + circulatory	Circulatory	Respiratory	Pneumonia and influenza	Circulating types of influenza^b^
2015/2016	Age groups Excess mortality per 100,000 population (95% CI)						A(H1N1)pdm09 (61%) Other A (5%) B/Victoria (34%) ___
	0–64	5.4 (4.8; 6.1)	3.2 (2.9; 3.5)	1.6 (1.3; 1.9)	1.6 (1.4; 1.7)	0.9 (0.8; 1.0)	
	65–74	33.3 (26.0; 40.6)	12.2 (8.2; 16.2)	7.3 (4.0; 10.6)	4.9 (3.2; 6.7)	3.3 (2.6; 4.0)	
	≥75	160.8 (122.9; 199.0)	64.3 (42.1; 86.8)	32.0 (17.2; 47.1)	29.9 (20.0; 39.9)	2.8 (0.2; 5.4)	
	All ages	22.9 (18.9; 26.9)	9.8 (7.5; 12.1)	5.0 (3.5; 6.6)	4.6 (3.6; 5.5)	1.3 (1.0; 1.6)	
2016/2017	Age groups Excess mortality per 100,000 population (95% CI);						A(H3N2) (94%) A (not subtyped) (5%)
	0–64	4.8 (4.1; 5.5)	2.0 (1.7; 2.3)	1.1 (0.8; 1.4)	0.9 (0.7; 1.0)	0.4 (0.3; 0.5)	
	65–74	43.7 (36.6; 50.8)	23.7 (19.8; 27.6)	11.1 (7.8; 14.5)	12.7 (10.9; 14.4)	5.0 (4.3; 5.7)	
	≥75	473.7 (437.0; 510.0)	269.2 (247.8; 290.4)	121.1 (107.0; 135.1)	145.1 (135.2; 155.0)	47.4 (44.5; 50.3)	
	All ages	52.9 (45.2; 52.2)	29.3 (24.9; 28.9)	13.4 (11.0; 13.7)	15.6 (13.4; 15.2)	5.3 (4.6; 5.1)	

Denmark							
Season	Cause of death	All‐cause	Respiratory + circulatory	Circulatory	Respiratory	Pneumonia and influenza	Circulating types of influenza^c^
2015/2016	Age groups Excess mortality per 100,000 population (95% CI)						A(H1N1)pdm09 (51%) A(H3N2) (5%) B/Victoria (44%) ___
	0–64	8.9 (6.7; 11.2)	2.6 (1.7; 3.5)	0.1 (−0.6; 0.9)	2.4 (2.0; 2.9)	0.6 (0.3; 0.9)	
	65–74	−17.5 (−34.5; −0.3)	11.4 (2.2; 20.6)	5.2 (−2.4; 12.8)	6.2 (0.8; 11.7)	0.4 (−1.7; 2.6)	
	≥75	191.1 (140.1; 243.0)	78.8 (47.3; 110.9)	54.6 (29.8; 79.7)	22.2 (6.4; 38.3)	13.7 (4.3; 23.4)	
	All ages	20.3 (15.8; 25.0)	9.4 (6.4; 12.3)	4.8 (2.5; 7.1)	4.4 (2.9; 6.0)	1.6 (0.8; 2.3)	
2016/2017	Age groups Excess mortality per 100,000 population (95% CI)						A(H3N2) (97%) A(H1N1)pdm09 (1%) Mixed B (2%)
	0–64	4.2 (2.1; 6.3)	−0.4 (−1.3; 0.5)	−1.1 (−1.9; −0.3)	0.8 (0.3; 1.2)	−0.3 (−0.5; −0.1)	
	65–74	17.5 (1.5; 33.6)	10.3 (1.1; 19.5)	−0.3 (−7.6; 7.1)	10.2 (4.8; 15.6)	1.5 (−0.9; 3.9)	
	≥75	237.1 (185.3; 289.6)	149.2 (116.7; 182.1)	82.4 (57.4; 107.5)	62.7 (46.3; 79.5)	9.5 (0.3; 19.1)	
	All ages	24.0 (19.3; 28.7)	12.3 (9.3; 15.4)	5.4 (3.1; 7.7)	6.6 (5.0; 8.2)	0.7 (−0.1; 1.5)	

*Note*: The preceding seasons have not been included as they were used for generating the baseline estimates. Furthermore, the columns for each age group do not add up to the all‐cause excess; this is to be expected, as they are separate models with separate baselines.

^a^ Morbidity and Mortality Weekly Reports.^24^, ^25^

^b^ Centro Nacional de Epidemiología, Instituto de Salud Carlos III.^26^, ^27^

^c^ Statens Serums Institut report.^28^, ^29^

**TABLE 2:** Ratio between cumulated pneumonia and influenza excess mortality and cumulated respiratory, circulatory, respiratory and circulatory, and all‐cause excess mortality during the winter season (Week 40 to Week 20), based on the EuroMOMO model, for the 2015/2016 and 2016/2017 seasons, in the United States, Spain, and Denmark

The United States						
Season	Cause of death	Pneumonia and influenza	Respiratory	Circulatory	Respiratory and circulatory	All‐cause
2015/2016	Age groups					
	0–64	1.0	1.95	6.32	8.55	23.82
	65–74	1.0	1.34	3.60	4.90	4.96
	≥75	1.0	1.86	−4.54	−2.82	−3.13
	All ages	1.0	1.72	1.93	3.67	10.17
2016/2017	Age groups					
	0–64	1.0	−14.50	−64.00	−80.50	−245.50
	65–74	1.0	2.68	3.34	5.99	10.81
	≥75	1.0	1.96	1.33	3.28	5.98
	All ages	1.0	2.18	2.26	4.45	9.24

Spain						
Season	Cause of death	Pneumonia and influenza	Respiratory	Circulatory	Respiratory and circulatory	All‐cause
2015/2016	Age groups					
	0–64	1.0	1.78	1.78	3.56	6.00
	65–74	1.0	1.48	2.21	3.70	10.09
	≥75	1.0	10.68	11.43	22.96	57.43
	All ages	1.0	3.54	3.85	7.54	17.62
2016/2017	Age groups					
	0–64	1.0	2.25	2.75	5.00	12.00
	65–74	1.0	2.54	2.22	4.74	8.74
	≥75	1.0	3.06	2.55	5.68	9.99
	All ages	1.0	2.94	2.53	5.53	9.98

Denmark						
Season	Cause of death	Pneumonia and influenza	Respiratory	Circulatory	Respiratory and circulatory	All‐cause
2015/2016	Age groups					
	0–64	1.0	2.25	2.75	5.00	12.00
	65–74	1.0	2.54	2.22	4.74	8.74
	≥75	1.0	3.06	2.55	5.68	9.99
	All ages	1.0	2.94	2.53	5.53	9.98
2016/2017	Age groups					
	0–64	1.0	−2.42	3.55	1.23	−13.61
	65–74	1.0	6.89	−0.20	6.95	11.85
	≥75	1.0	6.58	8.66	15.67	24.91
	All ages	1.0	9.51	7.86	17.84	34.75

*Note*: The ratios are based on the corresponding excess mortality estimates in Table 1.

### 3.5 Seasonal all‐cause and cause‐specific excess mortality—Spain

For all ages, the mean excess mortality for the two seasons based on all‐cause, respiratory and circulatory, circulatory, respiratory, and P&I was 37.9, 19.6, 9.2, 10.1, and 3.3 per 100,000 population, respectively (Table 1). These mean estimates corresponded to 16,197, 8347, 3935, 4300, and 1414 deaths, respectively. The excess estimates for the 2016/2017 season were higher than those for the 2015/2016 season. Influenza A (H1N1)pdm09 viruses predominated during the 2015/2016 season, whereas influenza A(H3N2) predominated during the 2016/2017 season (Table 1). The ratios between P&I excess mortality and the excess mortality of the respective causes of death were all positive but decreased from the 2015/2016 to the 2016/2017 season (Table 2). The range in ratios for Spain were larger than in the United States; for example, all‐cause excess deaths were 10–18 times more each year than P&I excess deaths. For all age groups, the ratios were positive across each of the cause of death categories and was highest when compared with all‐cause deaths.

### 3.6 Seasonal all‐cause and cause‐specific excess mortality—Denmark

For all ages, the mean excess mortality during the 2015/2016 and 2016/2017 seasons based on all‐cause, respiratory and circulatory, circulatory, respiratory, and P&I was 22.16, 10.84, 5.12, 5.5, and 1.3 per 100,000 population, respectively (Table 1). These mean estimates corresponded to 1261, 617, 291, 314, and 64 deaths, respectively. The excess mortality estimates were similar across the two seasons except for P&I excess mortality, which decreased by a factor of two in 2016/2017. Influenza A (H1N1)pdm09 viruses predominated during the 2015/2016 season, whereas influenza A(H3N2) predominated during the 2016/2017 season (Table 1). The ratios between all‐cause excess P&I mortality and the excess mortality from the respective cause‐specific other causes of death were all positive, and all increased from the 2015/2016 to the 2016/2017 season. The range in ratios for Denmark was larger than in the United States; for example, all‐cause excess deaths were 13–35 times more each year than P&I excess deaths. The ratios were not consistently positive across age groups (Table 2).

## 4 DISCUSSION

We found that the periods of weekly excess mortality for all‐cause and the respective cause‐specific causes of death were generally similar with respect to when the excess periods started, their length, and their timing of peaks, especially for the United States and Spain. Furthermore, we found that these periods were within the period where the influenza season typically occurs (Week 40 to Week 20 of the following year).

The ratios between cumulative seasonal all‐cause excess mortality and excess mortality of the cause‐specific causes of death (except for P&I) remained constant for Denmark across the two seasons. However, for the United States, a constant ratio was only observed for P&I mortality, whereas for Spain, the constant ratio was only observed for circulatory excess mortality. These ratios did not remain constant when stratified by age groups. Overall, this suggests that excess mortality estimates based on all‐cause may be used to infer excess mortality estimates for some of the cause‐specific categories only, but these inferences may only work within a country as the ratios vary substantially between nations.

Our seasonal all‐cause excess mortality estimates for the United States for influenza seasons 2015/2016 and 2016/2017 were 15,068 deaths (10,582–19,558) and 46,292 deaths (42,047–50,540), respectively. The estimates for season 2015/2016 were lower than the estimates published by the Centers for Disease Control and Prevention (CDC), whereas the estimates for season 2016/2017 fell within the range of the estimates published by CDC of 23,000 deaths (17,000–35,000) for season 2015/2016 and 38,000 deaths (29,000–61,000) for season 2016/2017.^30^ Our seasonal respiratory and circulatory excess mortality estimates for season 2015/2016 and season 2016/2017 were substantially lower at 5425 (2980–7873) and 22,306 (20,173–24,441), respectively. The CDC estimates influenza‐associated deaths based on the number of laboratory‐confirmed influenza‐associated hospitalizations, which have been adjusted for under‐detection of influenza. This is done by using a death to hospitalization ratio that represents the expected number of influenza deaths relative to the number of influenza‐associated hospitalizations.^7^, ^30^ In principle, using laboratory‐confirmed influenza‐associated hospitalizations adjusted for the proportion of individuals who may not be tested for influenza may be an accurate way of approximating the true underlying mortality burden of influenza. In contrast, estimates based on all‐cause excess mortality are expected to be an overestimate or, perhaps, an upper bound of the true underlying mortality burden.^8^ Given this, it is surprising that our all‐cause excess mortality estimates were not considerably higher than the CDC estimates. One potential explanation for this might have been that we did not truncate negative excess values, such that any week where the predicted number of excess deaths was lower than the expected number of deaths those deaths were subtracted from total excess deaths. However, as shown in Figure 1, the seasons we examined did not have periods where the observed mortality was considerably below the baseline. Furthermore, we generated seasonal estimates where negative values had been truncated (22,558 deaths and 48,421 deaths for the seasons 2015/2016 and 2016/2017, respectively). These estimates fell within the ranges of the estimates published by the CDC.

Our seasonal all‐cause excess mortality estimates for Denmark for the 2015/2016 and 2016/2017 seasons were 20.3 (15.8–25.0) and 24.0 (19.3–28.7) per 100,000 population. They were around twice as high as the influenza mortality estimates published by the State Serum Institute (SSI) of 8.3 (7.1–9.5) and 13.04 (11.75–14.38) per 100,000 population, respectively.^31^ Our seasonal respiratory and circulatory excess mortality estimates at 9.4 (6.4–12.3) and 12.3 (9.3–15.4) per 100,000 population for the 2015/2016 and the 2016/2017 seasons were in agreement with the national estimates. SSI estimates influenza‐associated deaths based on the FluMOMO model, which is a multivariable time series model with all‐cause mortality as outcome and influenza activity and extreme temperatures as explanatory variables while adjusting for time trend and seasonality.^6^ Although influenza may lead to excess mortality in causes of death not contained in the respiratory and circulatory categories, it is not surprising that the estimates based on these two categories came closer to the official estimates, which includes laboratory‐confirmed indicators of influenza‐activity, than the estimates based on all‐cause mortality.^1^, ^8^, ^12^

Our seasonal all‐cause excess mortality estimates for Spain for the 2015/2016 and 2016/2017 seasons were 22.9 (18.9–26.9) and 52.9 (49.1–56.8) per 100,000 population. They were around 3.5 and 1.5 times higher than the influenza mortality estimates reported by the CNE‐ISCIII of 6.0 (5.4–6.6) and 30.6 (29.5–31.8) per 100,000 population, respectively.^32^ Our seasonal respiratory and circulatory excess mortality estimates were more similar at 9.8 (7.5–12.1) and 29.3 (27.1–31.4) per 100,000 population for the 2015/2016 and the 2016/2017 seasons, respectively. Like SSI, CNE‐ISCIII uses the FluMOMO model to estimate influenza‐associated mortality.

It is unclear whether there is a constant proportionality between the P&I deaths and other cause‐specific or all‐cause excess mortality estimates. In all three countries, proportionality differed by season. The differences were smallest in the United States, perhaps owing to the larger population, which may provide more year‐to‐year stability. For example, for every one excess death due to P&I there were 9–10 times more all‐cause excess deaths, 10–18 times more all‐cause deaths in Spain and 13–35 times more all‐cause deaths in Denmark compared to P&I deaths. This proportionality between P&I and all‐cause excess mortality observed in the United States is consistent with the linear correlation observed by Simonsen et al, although their model suggested that P&I excess mortality made up around 25% of all‐cause excess mortality.^33^ In addition to population size, differences in coding practices, underlying diseases in the population, and the circulation of different influenza type or subtype viruses may also be an important part of the explanation. Furthermore, vaccination programs in different risk groups likely vary between countries. Although the pattern of circulating influenza types was similar between the countries, the vaccination programs, underlying immunity, and contact patterns in the population may vary and result in differences in excess mortality. It would be interesting to explore the proportionality we have observed, in particular for Denmark, within the respective countries. If this proportionality withstands further scrutiny, it may allow countries to approximate cause‐specific excess mortality, for example, respiratory excess mortality, based on all‐cause excess mortality and thus obtain timely cause‐specific excess mortality estimates.

Our approach did have limitations. The EuroMOMO model was originally designed for all‐cause mortality data used in near real time. Furthermore, it does not include indicators of influenza‐activity, which may lead to residual confounding in the form of attributing winter excess mortality, that is, in fact, due to other factors (such as other respiratory viruses and temperature) than influenza.^8^, ^9^, ^10^ Finally, it is worth emphasizing the fact that we only analyzed data from three relatively similar countries in that they are all high‐income countries with temperate climates.

This analysis does not settle the discussion on the differences and merits of using all‐cause mortality data and cause‐specific mortality data for estimating mortality related to influenza as it is notoriously difficult and purposes with the estimations may differ.^13^ On the one hand, all‐cause excess mortality estimates can be made available weekly in many countries and seem to display a weekly pattern that suggests that all‐cause excess mortality move proportionally to excess mortality of causes of death that is closely related to influenza (such as respiratory causes of death) within the periods typically associated with influenza. Furthermore, our all‐cause excess mortality estimates approximated the influenza mortality burden estimates published by CDC, which are based on laboratory‐confirmed hospitalizations.^7^, ^30^ On the other hand, the proportionality between the seasonally cumulated all‐cause excess mortality and the select cause‐specific excess mortality estimates (such as P&I and respiratory) does not seem to be reliably constant across seasons within all three countries. Additionally, all‐cause excess mortality estimates come with the risk of losing specificity and consequently overestimating the influenza mortality burden. This was indicated by the fact that our estimates for Denmark and Spain were between 1.5 and 3.5 times higher than the estimates produced by the FluMOMO model, which includes indicators of influenza‐activity and temperature, whereas our respiratory and circulatory excess mortality estimates approximated the FluMOMO estimates.^6^, ^31^, ^32^ Moreover, some cause‐specific mortality data are increasingly available more quickly in some countries; for example, CDC has access to P&I mortality data on a weekly basis, and additional advances in the timeliness of data availability are likely to be gained from the ongoing pandemic.^4^ However, it is important to note that these real‐time cause‐specific data may not be clean and the death counts are continuously updated as deaths are reported.

In conclusion, using a simple model of all‐cause excess mortality is a valuable tool for timely risk assessment of seasonal influenza and emerging threats such as the COVID‐19 pandemic, as the data are readily available in many countries, and the approach is not sensitive to coding practices in cause‐of‐death‐registers and collection of other indicators. To obtain precise estimates of excess mortality related to influenza, all‐cause mortality data should be supplemented with cause‐specific data and indicators of influenza transmission.

## AUTHOR CONTRIBUTIONS

**Sebastian Sudergaard Schmidt:** Conceptualization; formal analysis; methodology. **Angela Iuliano:** Data curation; formal analysis; methodology; supervision. **Lasse Vestergaard:** Data curation; supervision. **Clara Mazagatos‐Ateca:** Data curation. **Amparo Larrauri:** Data curation. **Sonja Olsen:** Conceptualization; methodology; supervision. **Jens Nielsen:** Conceptualization; methodology; supervision. **Joshua A. Salomon:** Conceptualization; methodology. **Tyra Krause:** Conceptualization; methodology.

## DISCLAIMER

The findings and conclusions in this report are those of the authors and do not necessarily represent the official position of the Centers for Disease Control and Prevention.

### PEER REVIEW

The peer review history for this article is available at https://publons.com/publon/10.1111/irv.12966.

## Data Availability

The data utilized in this manuscript are not publicly available given the identifiable nature of some of the components of these data. However, a limited variable data for the United States are available here: https://wonder.cdc.gov/Deaths-by-Underlying-Cause.html. Limited death data for Denmark are available here: https://www.dst.dk/en/Statistik/emner/borgere/befolkning/doedsfald. Limited information on deaths for Spain are located here: https://ine.es/dyngs/INEbase/en/operacion.htm?c=Estadistica_C&cid=1254736176780&menu=ultiDatos&idp=1254735573175.

## ENDNOTE

* ISO‐weeks is a widely leap week calendar system where each year has either 52 or 53 full weeks. It was issued by the International Organization for Standardization and has been adopted by the European Committee for Standardization.
